# Next generation sequencing profiling identifies miR-574-3p and miR-660-5p as potential novel prognostic markers for breast cancer

**DOI:** 10.1186/s12864-015-1899-0

**Published:** 2015-09-29

**Authors:** Preethi Krishnan, Sunita Ghosh, Bo Wang, Dongping Li, Ashok Narasimhan, Richard Berendt, Kathryn Graham, John R. Mackey, Olga Kovalchuk, Sambasivarao Damaraju

**Affiliations:** Department of Laboratory Medicine and Pathology, University of Alberta, 11560-University Avenue, Edmonton, AB T6G 1Z2 Canada; Department of Oncology, University of Alberta, Edmonton, AB Canada; Cross Cancer Institute, Edmonton, AB Canada; Department of Biological Sciences, University of Lethbridge, Lethbridge, AB Canada

**Keywords:** microRNA, Next generation sequencing, Breast cancer, Prognostic marker, miR-574-3p, miR-660-5p, Reduction mammoplasty, Overall survival, Recurrence free survival, TCGA

## Abstract

**Background:**

Prognostication of Breast Cancer (BC) relies largely on traditional clinical factors and biomarkers such as hormone or growth factor receptors. Due to their suboptimal specificities, it is challenging to accurately identify the subset of patients who are likely to undergo recurrence and there remains a major need for markers of higher utility to guide therapeutic decisions. MicroRNAs (miRNAs) are small non-coding RNAs that function as post-transcriptional regulators of gene expression and have shown promise as potential prognostic markers in several cancer types including BC.

**Results:**

In our study, we sequenced miRNAs from 104 BC samples and 11 apparently healthy normal (reduction mammoplasty) breast tissues. We used Case–control (CC) and Case-only (CO) statistical paradigm to identify prognostic markers. Cox-proportional hazards regression model was employed and risk score analysis was performed to identify miRNA signature independent of potential confounders. Representative miRNAs were validated using qRT-PCR. Gene targets for prognostic miRNAs were identified using *in silico* predictions and in-house BC transcriptome dataset. Gene ontology terms were identified using DAVID bioinformatics v6.7. A total of 1,423 miRNAs were captured. In the CC approach, 126 miRNAs were retained with predetermined criteria for good read counts, from which 80 miRNAs were differentially expressed. Of these, four and two miRNAs were significant for Overall Survival (OS) and Recurrence Free Survival (RFS), respectively. In the CO approach, from 147 miRNAs retained after filtering, 11 and 4 miRNAs were significant for OS and RFS, respectively. In both the approaches, the risk scores were significant after adjusting for potential confounders. The miRNAs associated with OS identified in our cohort were validated using an external dataset from The Cancer Genome Atlas (TCGA) project. Targets for the identified miRNAs were enriched for cell proliferation, invasion and migration.

**Conclusions:**

The study identified twelve non-redundant miRNAs associated with OS and/or RFS. These signatures include those that were reported by others in BC or other cancers. Importantly we report for the first time two new candidate miRNAs (miR-574-3p and miR-660-5p) as promising prognostic markers. Independent validation of signatures (for OS) using an external dataset from TCGA further strengthened the study findings.

**Electronic supplementary material:**

The online version of this article (doi:10.1186/s12864-015-1899-0) contains supplementary material, which is available to authorized users.

## Background

The global burden of breast cancer (BC) is 1.7 million and is one of the leading causes of cancer related death among women and the most frequently diagnosed cancer in 140 of 182 countries, as per the 2012 statistics [1]. Although advancements in diagnosis, screening and awareness help identify BC at an early stage, optimal management has remained a challenge due to its histological and molecular heterogeneity [2], and varying response to therapies even within clinical subtypes of BC [3]. Identification and validation of prognostic markers that can stratify patients based on their risk for recurrence and/or death may help in optimizing therapies to improve disease outcomes and quality of life. Estrogen Receptor (ER) and Human Epidermal Growth Factor Receptor 2 (HER2) are widely being used as both prognostic and predictive markers but remain as imperfect estimators of the risk for recurrence [4]. While, messenger RNA (mRNA) signatures from global gene expression profiling have also been put forth as potential prognostic markers for BC [5–8], their utility is limited to specific clinical settings [9]. This further emphasizes the need to identify robust prognostic markers with higher sensitivity, accuracy and reproducibility.

MicroRNAs (miRNAs, 18–25 nt) are evolutionarily conserved small non-coding RNAs that have shown promise as both diagnostic and prognostic biomarkers for several cancer types [10]. Predominantly, miRNAs behave as post-transcriptional regulators of gene expression, promoting either mRNA degradation or translation inhibition, depending upon the complementarity shared between the seed sequence of miRNAs and the corresponding 3' untranslated region of the target sequence [11–13]. However, studies have shown that they also activate gene expression [14]. Being either pleiotropic (one miRNA regulating several mRNAs) or highly redundant (several miRNAs targeting one mRNA) in nature [15], the impact of miRNA dysregulation in cancer is complex and yet promising in the overall landscape of tumorigenesis and prognostication.

Although several studies have highlighted the significance of miRNAs as diagnostic [16, 17] and prognostic markers for various cancers [18, 19], including BC [20–23], a consensus signature has not yet been identified due to differences in the profiling platforms employed, analytical approaches implemented, sample types (e.g. adjacent normal tissues or reduction mammoplasty specimens) used for analysis and tumor heterogeneity. The majority of the studies have utilized profiling platforms such as microarray or qRT-PCR, which are limited to the detection of known targets at the time of assay development. Hybridization platforms are also burdened with the problems of cross hybridization, background signal, low sensitivity and limitations on the dynamic range of detection. These problems are now overcome by Next Generation Sequencing (NGS) platforms [24]. NGS also offers the advantage of capturing not just miRNAs but a whole repertoire of small RNAs, even those present in low abundance [25], thus enabling a comprehensive analysis of small RNAome. However, despite several advantages offered by NGS, only few studies have utilized NGS platform to identify prognostic markers for BC [26, 27].

Statistical methods implemented in a study also play a vital role in determining the reproducibility of findings in a prognostic signature. Two methods to identify prognostic markers are widely used in the published literature– the case–control (CC) approach [20, 23] and the case-only (CO) approach [18, 19, 28]. While the former method utilizes a set of differentially expressed miRNAs for downstream analysis, the latter offers the advantage of being unbiased in selecting miRNAs for further analysis. Although each of the methods has been used in published miRNA studies, no study has analyzed a dataset using both the methods to compare and identify the best approach.

**Fig. 1:**
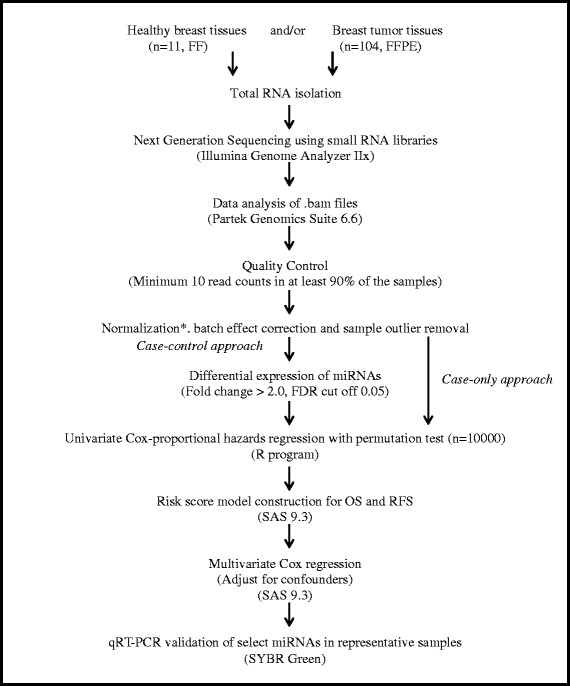
Overall Workflow of the study. In literature, there are two widely used approaches to identify RNAs with prognostic significance – Case–control approach and Case only. In the former approach, only differentially expressed (DE) RNAs are considered for survival analysis whereas in the latter approach, all of the profiled RNAs are considered for survival analysis which therefore aids in identifying prognostic RNAs which would have otherwise not been identified in case–control. While either of the two approaches has been adopted in literature, both the approaches have been followed in this study. FF = Fresh Frozen; FFPE = Formalin Fixed Paraffin Embedded; Normalization* = Reads per kilobase per million (RPKM); FDR = False Discovery Rate; OS = Overall Survival; RFS = Recurrence Free Survival

In this study, we hypothesized that relative variations in miRNA expression in tumors and/or apparently normal (non-malignant) tissues contribute to inter-individual differences in disease trajectory and eventual treatment outcomes. We profiled miRNAs from 104 breast cancers, predominantly of Luminal A and triple negative subtypes and 11 normal tissues (reduction mammoplasty specimens) using the NGS platform (Table 1). Our choice of reduction mammoplasty specimens from apparently healthy subjects was based on the recent literature evidence that comparisons between reduction mammoplasty specimens and tumor adjacent normal tissues showed differential expression of miRNAs, suggesting that adjacent normal tissue may not mirror the normal tissue in terms of histological and molecular characteristics [29]. Profiling of long non-coding RNAs showed differences between tumor adjacent normal tissues and tumor tissues. Differentially expressed long non-coding RNAs were not identified from tumor adjacent normal tissues vs. tumor tissues but were identified only when tumor tissues were compared with reduction mammoplasty specimens, further suggesting that reduction mammoplasty specimens may be the optimal baseline tissue [30]. Our specific objectives were as follows: (i) to identify differentially expressed miRNAs in breast tissues (normal vs. tumor tissues) and (ii) to identify miRNAs as prognostic markers (outcome: Overall Survival, OS and Recurrence Free Survival, RFS) for BC (Fig. 1) and validate the signatures using an external dataset. We have identified a total of twelve miRNAs associated with OS and/or RFS for BC. Of these twelve, we have replicated the prognostic significance of ten miRNAs already reported in literature for BC. To the best of our knowledge, this is the first study to report two novel miRNAs (miR-574-3p and miR-660-5p) for BC prognosis.

## Results

### Descriptive statistics of NGS and differentially expressed miRNAs

**Fig. 2:**
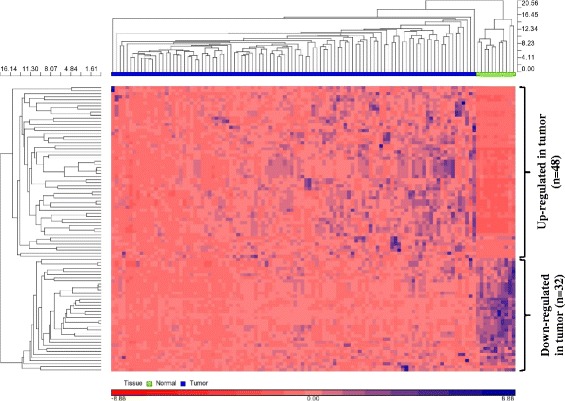
Unsupervised hierarchical clustering (HC) using differentially expressed miRNAs. Unsupervised hierarchical clustering of 80 differentially expressed miRNAs was performed using Euclidean as distance measure and Average linkage method for linkage analysis. HC shows normal and tumor tissues as distinct clusters. 48 miRNAs were up-regulated in tumor and 32 miRNAs were down-regulated in tumor relative to normal tissues. Rows represent miRNAs and columns represent samples

A total of 164,237,348 reads and 10,016,964 reads were detected from the tumor and normal samples, respectively, of which 59 % and 51 % of the reads were retained after adapter trimming. 84–87 % of the reads were mappable to the reference human genome and a total of 25,352,720 reads were mappable to different non-coding RNA classes (miRNAs, piRNAs, snoRNAs, snRNAs, rRNAs and tRNAs). A total of 25,003,223 reads mappable to mature human miRNAs belonged to 1,423 unique miRNAs (RNAs with at least one read count in any of the samples). A total of 126 miRNAs were retained after filtering for low read counts. Following normalization and batch effects correction (Additional file 1: Figure S1), one tumor sample was identified as a potential outlier and was removed from further analysis. Of the 126 miRNAs, 80 were differentially expressed (DE) with fold change (FC) > 2.0 and false discovery rate (FDR) cut-off of 0.05, of which 48 miRNAs were up-regulated and 32 were down-regulated (Additional file 2: Table S1). Unsupervised hierarchical clustering was performed using DE miRNAs. As expected, there was a clear separation of normal and tumor samples, illustrating that the generated miRNA signatures differentiate the two tissue types (Fig. 2). Also, the clustering of samples based on DE is to indicate that the samples are differentiated by the relative expression of a common set of miRNAs rather than by unique miRNAs.

### miRNAs as prognostic signatures for OS and RFS

**Fig. 3:**
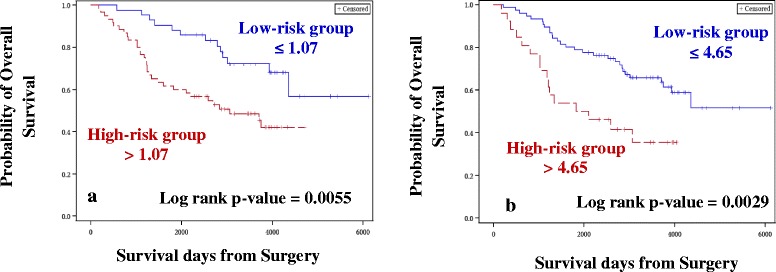
Kaplan-Meier plots for Overall Survival (Discovery cohort). Kaplan-Meier plots were used to estimate OS in Case–control approach (**a**) and Case-only approach (**b**). Log rank test was performed to assess differences in survival between the two risk groups. Patients belonging to the high-risk group had shorter OS in both (**a**) and (**b**)
- *Case-only approach*: One hundred and forty seven miRNAs retained after filtering for low read counts were treated as continuous variables and subjected to univariate Cox analysis followed by the permutation test. In this analysis, 11 miRNAs and 4 miRNAs were associated with OS (Table 2B) and RFS (Table 3B), respectively, and were used for constructing the risk score. A risk score cut-off point of 4.65 for OS was used to dichotomize the cases into low- (≤4.65) and high-risk groups (>4.65). Similarly, samples were grouped into two risk groups, based on the cut-off point estimated for RFS (1.17). Risk score was then treated as a categorical variable and entered into the univariate Cox model. Similar to the case–control approach, the higher-risk group was found to have both shorter OS (HR = 2.76, *p* = 0.002; Table 4B, Fig. 3b) and RFS (HR = 1.85, *p* = 0.02; Table 5B, Fig. 4b), after adjusting for confounders (tumor stage for OS and RFS).

**Fig. 4:**
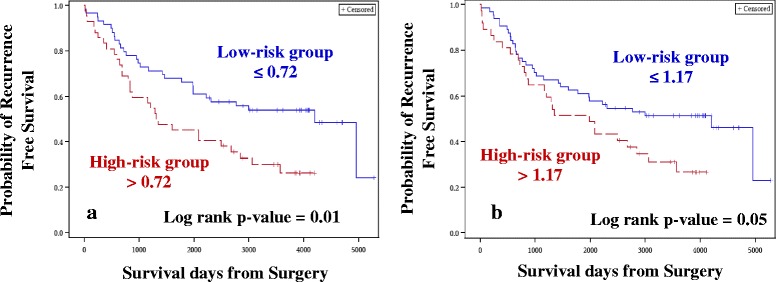
Kaplan-Meier plots for Recurrence Free Survival (Discovery cohort). Kaplan-Meier plots were used to estimate RFS in Case–control approach (**a**) and Case-only approach (**b**). Log rank test was performed to assess differences in survival between the two risk groups. Patients belonging to the high-risk group had shorter RFS in both (**a**) and (**b**)

- *Case–control approach:* Eighty DE miRNAs were treated as continuous variables and were subjected to univariate Cox analysis, followed by permutation test. Four miRNAs were associated with OS and two miRNAs were associated with RFS with permutation p ≤ 0.1. The four and two miRNAs identified for OS (Table 2A) and RFS (Table 3A), respectively were used for constructing the risk score. A risk score cut-off point of 1.07 for OS was used to dichotomize the cases into low- (≤1.07) and high-risk groups (>1.07). Similarly, samples were grouped into the two risk groups based on the cut-off point estimated for RFS (0.72). Risk score was then treated as a categorical variable and entered into the univariate Cox model. Tumor stage, grade, age at diagnosis and TNBC status were considered as other clinical covariates and were first tested for their significance in the univariate Cox model. Tumor stage, grade and age at diagnosis were considered as potential confounders, and, irrespective of their significance in the univariate analysis, they were entered into the multivariate model along with the risk score. The higher-risk group was found to have both shorter OS (Hazard ratio, HR = 2.71, *p* = 0.004; Table 4A, Fig. 3a) and RFS (HR = 2.27, *p* = 0.003; Table 5A, Fig. 4a), after adjusting for confounders (tumor stage and age at diagnosis for OS and tumor stage for RFS).

**Table 1:** Demographics of the samples chosen for the study

Characteristics	Discovery cohort	External validation cohort
	(*n* = 104)	(*n* = 84)
Median age at diagnosis in years (range)	50 (24 – 79)	54.5 (35 – 90)
Median follow up time from diagnosis in days (range)	2927.5 (170 – 6125)	1881.5 (174 – 3807)
Molecular subtypes		
Luminal A	62	51
Luminal B	2	0
Luminal B HER2	10	18
Triple Negative	30	15
Menopausal status		
Pre	37	24
Post	75	46
Peri	11	3
Unknown	1	11
Family history of Breast Cancer		
Yes	40	N/A
No	58	N/A
Unknown	6	N/A
*Stage*		
I	8	25
II	79	47
III	16	12
IV	1	0
Overall Grade		
Low	36	N/A
High	67	N/A
Unknown	1	N/A
*Vital Status*		
Alive	58	57
Dead	46	27
Relapse Status		
Relapse	61	N/A
No relapse	43	N/A
Treatment type		
Adjuvant	79	84
Neoadjuvant	25	0

N/A = Not available

**Table 2:** miRNAs significant for overall survival (Discovery cohort)

A. Case–control approach		
miRNA ID	Univariate Cox p-value	Permuted p-value
hsa-miR-15a-5p	0.02	0.03
hsa-miR-660-5p	0.03	0.04
hsa-miR-574-3p	0.08	0.07
hsa-miR-27a-3p	0.06	0.07
B. Case-only approach		
miRNA ID	Univariate Cox p-value	Permuted p-value
hsa-miR-210-3p	0.01	0.02
hsa-miR-15a-5p	0.02	0.03
hsa-miR-660-5p	0.03	0.04
hsa-miR-146b-5p	0.04	0.05
hsa-miR-374a-3p	0.04	0.05
hsa-miR-374a-5p	0.04	0.06
hsa-miR-27a-3p	0.06	0.07
hsa-miR-574-3p	0.08	0.07
hsa-miR-221-3p	0.07	0.08
hsa-miR-196a-5p	0.07	0.09
hsa-miR-425-5p	0.05	0.10

A: 80 miRNAs were differentially expressed with Fold change > 2.0 and at a FDR cut off <0.05. All 80 miRNAs were subjected to Univariate Cox proportional hazards regression and permutation test (*n* = 10,000) for Overall Survival (OS). Four miRNAs were significant for OS and were used to construct a risk score. Univariate Cox p-value is the unpermuted p-value for Univariate Cox model. B: All the miRNAs (*n* = 147) retained after filtering (minimum 10 read counts in at least 90 % samples) in cases were considered for further analysis. 11 miRNAs were significant for OS with permuted p-value ≤ 0.1 and were considered for constructing a risk score. Univariate Cox p-value is the unpermuted p-value for Univariate Cox model

**Table 3:** miRNAs significant for recurrence free survival (Discovery cohort)

A. Case–control approach		
miRNA ID	Univariate Cox p-value	Permuted p-value
hsa-miR-193b-3p	0.09	0.09
hsa-miR-15a-5p	0.08	0.10
B. Case-only approach		
miRNA ID	Univariate Cox p-value	Permuted p-value
hsa-miR-210-3p	0.01	0.02
hsa-miR-425-5p	0.05	0.08
hsa-miR-193b-3p	0.09	0.09
hsa-miR-15a-5p	0.08	0.10

A: 80 miRNAs were differentially expressed with Fold change > 2.0 and FDR cut off 0.05. All 80 miRNAs were subjected to Univariate Cox proportional hazards regression and permutation test (*n* = 10,000) for Recurrence Free Survival (RFS). Two miRNAs were significant for RFS with permutation p value ≤ 0.1 and were used for constructing risk score. Univariate Cox p-value is the unpermuted p-value for Univariate Cox model. B: All the miRNAs (*n* = 147) retained after filtering (minimum 10 read counts in at least 90 % samples) in cases were considered for further analysis. Four miRNAs were significant for RFS with permuted p-value ≤ 0.1 and were considered for constructing a risk score. Univariate Cox p-value is the unpermuted p-value for Univariate Cox model

**Table 4:** Univariate and Multivariate results for overall survival (Discovery cohort)

A. Case–control approach				
Parameter	Univariate analysis		Multivariate analysis	
	HR (95 % CI)	p-value	HR (95 % CI)	p-value
Risk score	2.44 (1.28 – 4.68)	0.01	2.71 (1.38 – 5.35)	0.004
Tumor stage	0.42 (0.22 – 0.81)	0.01	0.36 (0.18 – 0.74)	0.01
Tumor grade	1.93 (0.99 – 3.75)	0.05		
Age at diagnosis	1.05 (1.02 – 1.09)	0.003	1.04 (1.01 – 1.07)	0.02
TNBC status	0.88 (0.43 – 1.77)	0.71		
B. Case-only approach				
Parameter	Univariate analysis		Multivariate analysis	
	HR (95 % CI)	p-value	HR (95 % CI)	p-value
Risk score	2.48 (1.34 – 4.61)	0.004	2.76 (1.47 – 5.19)	0.002
Tumor stage	0.42 (0.22 – 0.81)	0.01	0.37 (0.19 – 0.72)	0.004
Tumor grade	1.93 (0.995 – 3.75)	0.05		
Age at diagnosis	1.05 (1.02 – 1.09)	0.003		
TNBC status	0.88 (0.43 – 1.77)	0.71		

A and B: The four and 11 miRNAs from Table 1A and B respectively were used to construct risk scores. Receiver Operating Characteristics Curve was used to dichotomize cases into low and high-risk groups. Univariate Cox proportional hazards regression model was run for risk score and for other clinical parameters. In the multivariate analysis, risk score was significant with p < 0.05 after adjusting for confounders.

*HR* Hazard Ratio; *CI* Confidence Interval; *TNBC* Triple Negative Breast Cancer

### qRT-PCR validations of miR-99b-5p, miR-574-3p, miR-769-5p and miR-660-5p

**Fig. 5:**
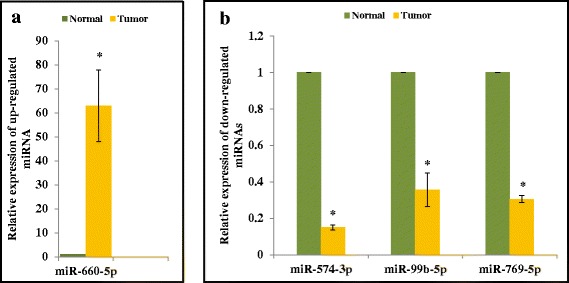
qRT-PCR validations of select miRNAs. **a** One up-regulated miRNA (miR-660-5p, FC = 12.8) was validated in a subset of samples (9 normal samples and 56 tumor samples). **b** Three down-regulated miRNAs (miR-574-3p, miR-99b-5p and miR-769-5p) were validated in a subset of samples (11 normal samples and 60 tumor samples). All the miRNAs were significantly (* = *p* < 0.05) differentially expressed, similar to the results obtained in NGS platform. miR-574-3p and miR-660-5p were also found to be associated with Overall Survival

The expressions of miR-99b-5p with a FC of −2.3, miR-574-3p with a FC of −5.8, miR-769-5p with a FC of −1.3 (down-regulated) and miR-660-5p with a FC of 12.8 (up-regulated) were tested in qRT-PCR to confirm the direction of effect and relative quantification agreement between NGS and qRT-PCR. Except for miR-660-5p, that was up-regulated (Fig. 5a), remaining three miRNAs were found to be significantly down-regulated in tumor tissues relative to normal samples. qRT-PCR experiments (Fig. 5b) supported the NGS findings.

### Identification of potential targets for miRNAs and their role in cancer biology

The in-house transcriptome (mRNA) datasets available for BC were accessed (GEO accession ID GSE22820) [31] and analyzed for DE of mRNAs from a matched subset of samples (*n* = 17). 2,869 genes (mRNAs) were DE, of which 628 were up-regulated and 2241 were down-regulated.

A combined total of 4,762 targets were predicted by TargetScan for the 12 miRNAs associated with OS and/or RFS. Of these, only 698 targets (~15 % of *in silico* predicted targets) overlapped with the mRNA expression dataset. This low percent overlap between *in silico* and *in situ* comparisons is expected when breast tissue specific expression signatures filtered for histological and molecular subtypes are used to interrogate the potential interactions between miRNA-mRNA. The profiled interactions with transcriptome data also serve as an approach for functional validation of the miRNA targets within breast tissues and minimize the number of false positive targets identified. A total of 168 clusters were found and when interrogated for gene ontology (GO) classifications with an enrichment score (ES) ≥ 1.3, 57 clusters were retained (Table 6). We identified two targets for miR-574-3p (DAB2IP and SAMD4A) which did not belong to any cluster due to limited hits. From the clusters, statistically significant GO terms (p < 0.05) related to cancer were identified. Specifically, the following terms were interrogated: transcription, blood vessel development, angiogenesis, cell growth, cell morphogenesis, cell motion, cell migration, cell signaling, mammary gland development, cell differentiation, cell proliferation, cell division and cytoskeletal organization. Targets of 8 out of 12 miRNAs (miR-15a-5p, miR-27a-3p, miR-374a-3p, miR-374a-5p, miR-221-3p, miR-196a-5p, miR-146b-5p and miR-660-5p) were enriched for any one of the above-mentioned terms (Additional file 3: Table S2). Targets of miR-574-3p, miR-425-5p, miR-210-3p and miR-193b-3p were clustered with an ES ≤ 1.3 when matched miRNA-mRNA data sets were used and were therefore not probed further. Similar analysis of identifying targets from DE mRNAs was attempted using unmatched samples from the same in-house BC dataset (*n* = 141). Excellent concordance was observed between the results (in terms of number of targets identified, GO terms and clusters) obtained from matched samples and unmatched samples (data not shown), indicating that the use of matched or unmatched samples have no profound impact on the identification of gene targets for the miRNAs.

**Table 5:** Univariate and multivariate results for recurrence free survival (Discovery cohort)

A. Case–control approach				
Parameter	Univariate analysis		Multivariate analysis	
	HR (95 % CI)	p-value	HR (95 % CI)	p-value
Risk score	1.95 (1.16 – 3.29)	0.01	2.27 (1.33 -3.88)	0.003
Tumor stage	0.42 (0.23 – 0.76)	0.01	0.34 (0.18 – 0.65)	0.001
Tumor grade	1.52 (0.88 – 2.63)	0.14		
Age at diagnosis	1.02 (0.99 – 1.05)	0.29		
TNBC status	0.75 (0.39 – 1.41)	0.37		
B. Case-only approach Parameter				
Parameter	Univariate analysis		Multivariate analysis	
	HR (95 % CI)	p-value	HR (95 % CI)	p-value
Risk score	1.68 (0.99 – 2.82)	0.05	1.85 (1.09 – 3.14)	0.02
Tumor stage	0.42 (0.23 – 0.79)	0.01	0.38 (0.20 – 0.71)	0.003
Tumor grade	1.52 (0.88 – 2.63)	0.14		
Age at diagnosis	1.02 (0.99 – 1.05)	0.29		
TNBC status	0.75 (0.39 – 1.41)	0.37		

A and B: The two and four miRNAs from Table 2A and B respectively were used to construct risk scores. Receiver Operating Characteristics Curve was used to dichotomize samples into low and high-risk groups. Univariate Cox proportional hazards regression model was run for risk score and for other clinical parameters. In the multivariate analysis, risk score was significant with p < 0.05 after adjusting for confounders.

*HR* Hazard Ratio; *CI* Confidence Interval; *TNBC* Triple Negative Breast Cancer

### Validation of OS-associated miRNAs in an external (TCGA) dataset

**Fig. 6:**
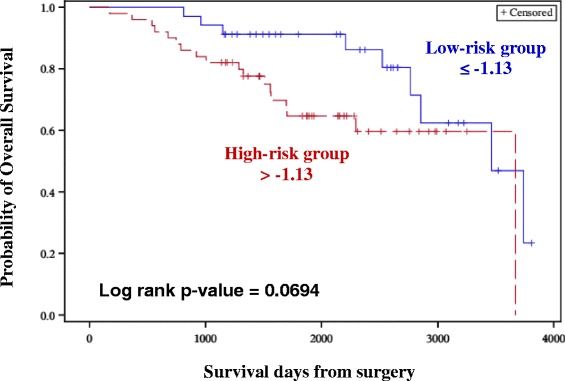
Kaplan-Meier plot for Overall Survival (External validation cohort). Kaplan-Meier plots were used to estimate OS in Case-only approach. Log rank test was performed to assess differences in survival between the two risk groups. Patients belonging to the high-risk group had shorter OS

Eleven miRNAs that were significant for OS in the CO approach were validated using an external dataset (The Cancer Genome Atlas, TCGA). Risk score was constructed using the eleven miRNAs. An optimal cut-off point was determined using ROC, to group samples into low (≤ −1.13) and high risk (> −1.13). Risk score which was considered as a categorical variable was significant with a p-value of 0.1 after adjusting for tumor stage. Similar to the discovery set, high risk group had shorter survival period with a HR of 2.07 (Fig. 6, Table 7).

**Table 6:** Identification of mRNA targets for miRNAs significant for OS and/or RFS

miRNA ID	Target Identification		Gene Ontology	
	Number of mRNA targets identified from TargetScan	Number of predicted targets overlapping with mRNA expression dataset	Total number of annotation clusters	Number of clusters with enrichment score ≥ 1.3
hsa-miR-15a-5p	1275	181	48	15
hsa-miR-27a-3p	1212	183	47	13
hsa-miR-193b-3p	222	30	4	0
hsa-miR-574-3p	13	2	0	0
hsa-miR-660-5p	149	25	2	1
hsa-miR-210-3p	32	6	1	0
hsa-miR-146b-5p	226	31	4	1
hsa-miR-374a-3p; hsa-miR-374a-5p	680	110	22	9
hsa-miR-221-3p	446	60	25	13
hsa-miR-196a-5p	295	46	13	5
hsa-miR-425-5p	212	24	2	0

Gene targets for 12 miRNAs significant in survival analysis were identified using *in silico* prediction (TargetScan) and were confirmed with in-house mRNA-miRNA matched expression dataset (*n* = 17). Gene ontology terms were identified for mRNAs overlapping with in-house dataset using DAVID bioinformatics tool. Only annotation clusters with Enrichment Score (ES) ≥ 1.3 were considered. However, no cluster with ES ≥ 1.3 could be identified for miR-193b-3p, miR-574-3p, miR-210-3p and miR-425-5p

## Discussion

In this study, we identified two miRNAs (miR-574-3p and miR-660-5p) as potential novel prognostic markers for BC, associated with OS. They have not been reported earlier for BC, for their association with either OS or RFS. Overall, from both the approaches (CC and CO) adopted for the study, eleven miRNAs and four miRNAs were significant for OS and RFS, respectively. Out of the four miRNAs identified for RFS, three miRNAs (miR-210-3p, miR-425-5p and miR-15a-5p) were also significant for OS.

In recent years, microRNAs have gained prominence as valuable prognostic markers for several cancer types, including BC. Although considerable progress has been made in this field, clinical application of these miRNAs as prognostic markers has not yet been possible because of the generation of different signatures by different studies with only a small number of overlapping molecules [9]. This discrepancy may be attributed to several reasons [32], the primary being the use of different profiling platforms. Studies employing hybridization techniques or qRT-PCR panels have generated signatures based on the available number of miRNAs. While there are ~2,588 miRNAs identified so far [33], as reported in the miRBase, only a few hundreds have been captured on these platforms [18, 34], of which even fewer have been detected in breast tissues. Therefore, we are blinded to the prognostic value of other miRNAs from the larger repertoire. On the other hand, NGS profiling of the entire miRNAome, including even the less abundant ones, can now be used to probe the larger repertoire, which was evident from our study. Approximately, breast tissue specific miRNAs comprise 55 % (*n* = 1,423) of the total miRNAs (*n* = 2,588 annotated thus far) and these were captured from the 11 normal breast tissues and 103 breast tumor tissues used for the study, which is by far the largest miRNA dataset to be interrogated for identifying prognostic markers for BC. It is also one of the largest studies in terms of number of samples with complete clinical information sequenced for BC.

### Statistical considerations

More often than not, high-throughput techniques suffer from the problems of high dimensionality (a higher number of markers but lower number of samples) and collinearity (correlation between two markers), leading to the generation of instable co-efficients in a traditional Cox-proportional hazards regression model [35]. In such cases, the inclusion of individual miRNAs to build a model may not yield reliable results, whereas considering miRNAs as continuous variables and constructing risk scores overcomes both these problems. However, there are two widely used methods (CC approach and CO approach) to identify miRNAs useful for constructing a risk score. In a typical CC approach, two groups are compared to identify DE miRNAs, which are assessed for their prognostic significance. A CO approach offers a wider data set for interrogation, allowing a comprehensive understanding of miRNAs. As a proof of principle, we adopted both the approaches to identify prognostic markers. As expected, higher numbers of miRNAs were identified as significant in the CO approach. Eleven miRNAs were significant for OS and four miRNAs were significant for RFS in the CO approach as opposed to four and two miRNAs significant for OS and RFS, respectively, in the CC method. miR-210-3p, miR-425-5p and miR-15a-5p were significant for both OS and RFS. A total of 12 non-redundant miRNAs were found to play a role in BC prognosis.

Overall, the differential expression in normal vs. tumor tissues and direction of effects show excellent agreement with what is known from published literature, as detailed below.

### Novel prognostic miRNAs for BC

Of the 12 miRNAs identified in this study, two miRNAs (miR-574-3p and miR-660-5p) are potential novel prognostic markers for BC. Both the miRNAs were DE in a tumor vs. normal comparison, with miR-574-3p being down-regulated (FC = −5.8) and miR-660-5p being up-regulated (FC = 12.8) in the tumor samples. A similar direction of effect has been observed for miR-574-3p and miR-660-5p for ovarian cancer [36], colorectal cancer [37] and gastric cancer [38]; and chronic lymphocytic leukemia [39], respectively. However, this is the first report of a potential prognostic role for these miRNAs in BC, although mechanistic insights are required to understand their contribution to tumorigenesis.

### miRNAs with dual roles as tumor suppressor and oncogene

In our study, miR-15a-5p was found to be up-regulated in breast tissues (FC = 12.16) and the same direction of expression was observed in Kaposi sarcoma [40] and papillary thyroid carcinomas [41]. However, in other cancer types such as colorectal cancer [42], non-small-cell lung cancer (NSCLC) [43] and pituitary tumors [44], it is expressed in the opposite direction (down-regulation). Amongst BC reports, Kodahl et al. have reported an up-regulation of this miRNA [45], and a recent report by Shinden et al. has shown miR-15a as an independent prognostic marker for BC [46]. Similarly, miR-27a-3p, which was found to be up-regulated in tumors (FC = 6.45) in our study, is in accordance with the direction of expression observed in pancreatic cancer [47] and glioma [48]. Tang et al. have also reported miR-27a to be an oncomiR, the high expression of which promotes breast tumor growth and metastasis and is associated with poor OS in BC patients [49]. However, it is down-regulated in bladder cancer, compared with the normal samples [50]. The observations on miR-15a-5p and miR-27a-3p point to the dual roles of an oncogene and a tumor suppressor and their relative role may be governed in a tissue-specific manner.

### miRNAs as oncogenes

We observed high expression (FC = 1.98) of miR-425-5p in breast tumors compared to the normal samples, which is concordant with the results published by Kodahl et al. for BC [45]. Likewise, Peng et al. have also observed the oncogenic function of miR-425, which promotes cell proliferation, cell cycle progression, migration and invasion in gastric cancer [51].

Up-regulation of miR-146b in tumors and its adverse effect on survival has been demonstrated in lung cancer [52, 53], thyroid carcinoma [54] and prostate cancer [55], among other cancer types. Interestingly, miR-146b-5p has also been reported to be up-regulated in BC, which is in accordance with our results (FC = 1.42) and is known to repress BRCA1 expression, thereby promoting cell proliferation [56].

miR-221 is a widely studied oncogene whose high expression is invariably associated with poor outcomes in several cancer types [57–59], including BC [60]. We also report the same direction of expression in tumor tissues with a FC of 1.27.

Cell proliferation, migration, invasion and metastasis have been found to be promoted in BC [61–63], glioblastoma [64, 65], head and neck cancer [66] and gastric cancer [67, 68] due to high expressions of miR-210, miR-196a and miR-374a (including miR-374a-3p and -5p), demonstrating their oncogenic potential. Their role as prognostic markers has also been studied in the above-mentioned cancer types. We were able to identify their prognostic significance following the CO approach, and these findings could have been missed if only the CC approach had been used. The read counts of the two groups (normal and tumor) revealed that these miRNAs were indeed present in higher amounts in tumors relative to the normal samples; the average read counts of miR-210-3p, miR-196a-5p, miR-374a-3p and miR-374a-5p in the normal samples were 2.5, 9.2, 0.7 and 1.09 respectively as against 59.7, 307.6, 46.1 and 108.9 for the tumor group. The lower read counts in normal samples have limited our ability to consider them in a CC study due to our stringent filtering criteria. Overall, the patterns of DE and prognostic significance for the above miRNAs mirror observations from other cancer types.

### miRNAs as tumor suppressors

In our study, apart from miR-574-3p, miR-193b-3p was also found to be down-regulated (FC = −4.3) in tumors compared to normal samples, which is in agreement with the studies on endometrioid adenocarcinoma [69], pancreatic cancer [70], oesophageal cancer [71] and gastric cancer [68]. Even in BC, Li et al. have reported a down-regulation of miR-193b in BC cell lines, and the low expression of miR-193b was found to be associated with shorter disease-free survival [72].

### Functional roles of the identified prognostic miRNAs

The prognostic significance for recurrence or survival of an associated miRNA is better appreciated from the aspect of potential functional impact on cellular signaling and metabolic pathways, as these contribute to cell death, invasion and overall outcomes for the patient. Apart from functional insights, the potential for development of therapeutics is also important. Keeping these factors in mind, the following discussion is focused on the delineation of pathways using GO terms that are specifically enriched by the identified prognostic miRNAs.

Databases such as TargetScan, miRanda (http://www.microrna.org/) and PicTar (http://pictar.mdc-berlin.de/) have predicted mRNA targets, but a validation of the predicted targets adds more credence to *in silico* predictions. To this end, we first predicted the targets for all 12 miRNAs using the commonly used database - TargetScan; these were then compared with DE mRNAs obtained from the in-house BC transcriptome dataset. GO terms were identified with a specific focus on terms pertaining to hallmarks of cancer. Interestingly, targets of eight miRNAs were found to be relevant for cell growth and development, indicating that these miRNAs may play key roles in tumorigenesis. Two targets (DAB2IP and SAMD4A) were found for miR-574-3p, of which DAB2IP is involved in apoptosis [73], cell survival [74], among other functions and SAMD4A functions as a translational regulator [75].

### Validation of the identified signatures

In a biomarker study, a validation of the findings across different platforms is critical to rule out technical artifacts. Four miRNAs exhibiting different FC (lowest FC being −1.3) were validated using qRT-PCR, with two of the representative miRNAs identified as significant in survival analysis. The validation of representative miRNAs confirms cross-platform concordance and the relative utility of the signatures identified. However, validations using independent cohorts are also crucial for a biomarker study as they facilitate inter-study concordance of expression trends and signatures. NGS data for BC with a larger sample size and complete clinical information are limited in the public domain. We used the available data from TCGA project and applied stringent filtering criteria to obtain a dataset that would be comparable to the discovery set. A total of eleven miRNAs which were found to be associated with OS from the CO approach were considered for validation using the TCGA dataset. TCGA samples were sequenced using Illumina Genome analyzer and Illumina HiSeq platforms. However, all samples were not sequenced on a single platform limiting the analysis and comparisons. Although our discovery set of samples were sequenced using the Illumina Genome analyzer, the number of samples with events (deaths, *n* = 8) on this platform were limited for the data from TCGA. Therefore, we considered samples sequenced using Illumina HiSeq with sufficient follow-up and events (deaths, *n* = 27). Due to the fact that NGS platform specific differences in read counts may potentially influence the risk scores we did not adopt the risk scores and cut-off points generated in discovery set. We therefore generated a new risk score for the validation set and an optimal cut-off point was estimated to dichotomize the samples into low and high risk groups. Multivariate analysis revealed that the risk score was significant with *p* = 0.1 after adjusting for tumor stage. TCGA dataset lacks information on tumor grade. However, tumor grade did not influence the multivariate analysis even in the discovery set. Therefore we reasoned that lack of information on grade in the TCGA data set would not have influenced the study findings. Although for our initial analysis using discovery set we considered p < 0.05 as nominal, the TCGA dataset did not meet this threshold, presumably due to modest sample size (*n* = 84) and events (*n* = 27) compared to the discovery set (sample size, *n* = 104 and events, *n* = 46). Nevertheless, we still observed the same direction of effect (Hazard Ratio), i.e., patients belonging to the high-risk group was associated with shorter survival period and this validates our initial observations from the discovery set.

Several differences existed between the discovery and validation datasets: (i) the NGS platform for discovery set was Genome Analyzer IIx where as for the validation set was HiSeq; (ii) the risk score cut-off point were estimated individually due to NGS platform differences; (iii) TCGA samples considered for this study were fresh frozen breast cancer tissues whereas the discovery set of breast cancer tissues were from FFPE blocks, (iv) information on tumor grade was not available for TCGA samples and (v) percent cellularity differences were also noted between the discovery and validation cohorts (see methods). However, despite these differences and other characteristics (Table 1), we could demonstrate the trends in the direction of effects (Hazard Ratio) in both the discovery and validation cohorts. The apparent lack of statistical significance (defined nominal value of 0.05) in the OS analysis attempted with TCGA data may be due to the limited sample size and limited number of events in the validation set affecting the power. Further validation of findings is warranted using independent cohorts and higher sample size and events. Overall, we report two novel miRNAs as potential prognostic markers for breast cancer. Remaining miRNAs reported in this study showed excellent concordance to the published reports for their role in BC prognosis.

**Table 7:** Univariate and multivariate results for overall survival (External Validation cohort/TCGA)

Parameter	Univariate analysis		Multivariate analysis	
	HR (95 % CI)	p-value	HR (95 % CI)	p-value
Risk score	2.16 (0.92 – 5.05)	0.08	2.07 (0.87 – 4.92)	0.101
Tumor stage	0.32 (0.13 – 0.78)	0.01	0.26 (0.1 – 0.67)	0.005
Age at diagnosis	1.03 (1.003 – 1.06)	0.03		
TNBC status	0.63 (0.19 – 2.12)	0.46		

The eleven miRNAs identified as significant for OS in CO approach from the discovery set was validated using TCGA dataset. Risk score was constructed using the 11 miRNAs and an optimal cut-off point was estimated using Receiver Operating Characteristics Curve, which dichotomized the samples into low and high-risk groups. Univariate Cox proportional hazards regression model was run for risk score and for other clinical parameters. In the multivariate analysis, risk score was significant with *p* = 0.1 after adjusting for tumor stage.

*HR* Hazard Ratio; *CI* Confidence Interval; *TNBC* Triple Negative Breast Cancer

## Conclusions

In summary, we identified a total of twelve non-redundant miRNAs associated with OS and/or RFS. As explained above, ten of the identified miRNAs have been reported in literature as associated with BC prognosis and lends support to the findings in this independent study. However, two miRNAs (miR-574-3p and miR-660-5p) have not been reported previously for BC prognosis. The use of NGS platform to profile miRNAs on a whole genome level in BC has been limited thus far in literature and the data provided complements such efforts towards a comprehensive search for biomarkers. The miRNAs reported for OS have also been validated in independent dataset (TCGA) and functional characterization may help to understand the complex interplay of miRNA mediated gene regulation.

Overall, despite the increasing feasibility of profiling miRNAs and their role in prognostication, mechanistic insights in to the role of miRNAs, establishing gold standard approaches for analysis, and confirmation of these findings by independent laboratories within the context of confounding variables (histological and molecular heterogeneity, stage, grade and treatment) are needed to advance these promising biomarkers into clinical validation.

There is also a growing body of evidence that other small non-coding RNAs such as tRNAs [76], snoRNAs [77] and piRNAs [78] may contribute to tumorigenesis; however their role in BC prognosis is an area of active investigation. Therefore, a deeper exploration of their roles may pave the way for a comprehensive understanding of the small non-coding RNA classes, aiding in the discovery of newer diagnostic and prognostic biomarkers for BC.

## Methods

### Discovery cohort

One hundred and four breast tumor samples (belonging to the invasive ductal carcinoma histological subtype), along with complete clinicopathological characteristics (Table 1), were accessed from the Alberta Cancer Research Biobank/Canadian Breast Cancer Foundation (CBCF) tumor bank (http://www.acrb.ca/). All of the samples sequenced were non-metastatic (M0) at the time of diagnosis except one, with metastatic cancer (M1) at the time of presentation. Of the 104 patients, 25 were treated with either standard chemotherapeutic drugs (neoadjuvant) or had undergone radiotherapy before tumor resection and 79 were given adjuvant therapy, and were treated predominantly with a polychemotherapy regimen of Taxotere, Adriamycin and Cyclophosphamide (TAC, *n* = 57). Despite standard care of therapy, 61 patients experienced recurrence and 43 did not; 46 patients had died and 58 were alive at the time of completion of this study. The median age was 50 (Range: 24–79) years and the median follow-up period was 2927.5 (Range: 170 – 6,125) days from the date of diagnosis (between years 1996 and 2008) till January 2015. Patients were further classified into different subtypes based on the presence or absence of immunohistochemical markers such as ER, progesterone receptor (PR) and HER2. Most of the samples (*n* = 62) belonged to Luminal A subtype with ER positive or PR positive and HER2 negative disease. Thirty samples were negative for ER, PR and HER2 based on immunohistochemical staining score and were classified as Triple Negative breast cancer (TNBC). Ten samples were positive for ER/PR and HER2 staining and were grouped into Luminal HER2 subtype. Two samples were positive for ER and PR (HER2 unknown), but the overall grade was high, and these were grouped into Luminal B subtype, as described earlier [79–81]. All the BC tissues were archived as Formalin Fixed Paraffin-Embedded (FFPE) tissue blocks. All of the samples considered for profiling of miRNAs showed ≥ 70 % tumor cells. The percent distribution of tumor cells in samples were as follows: 70 % (*n* = 7), 80 - 90 % (*n* = 13), 90 % (*n* = 24), 95 % (*n* = 35) and 100 % (*n* = 25). Eleven apparently normal breast tissues (confirmed by the pathologist to be free of malignancy, based on the absence of morphological and histological anomalies) were obtained from reduction mammoplasty surgery and were preserved as Fresh Frozen (FF) tissues.

We estimated the number of samples needed to detect statistically significant differences for the measured transcripts between cases and controls in the current study (http://bioinformatics.mdanderson.org/MicroarraySampleSize/ and http://linus.nci.nih.gov/brb/samplesize/) [82, 83]. The following parameters were considered to estimate the sample sizes: α = 0.05, β = 80 % and a fold difference of 2 or more in miRNA expression. Under these conditions, we needed at least 8–11 samples in each group (controls and cases). Our study design included 11 control samples and 104 cases, thus meeting the statistical rigor needed to interpret the data with confidence. Informed consent was obtained from all the patients and the study was approved by the local Institutional Research Ethics committee (Health Research Ethics board of Alberta- Cancer Committee).

### Total RNA isolation for small RNA sequencing

Briefly, FF tissues were homogenized using TRIzol (Invitrogen) and total RNA was isolated using Qiagen RNeasy kit according to manufacturers' instructions. Total RNA from FFPE blocks was isolated using the RecoverAll Total Nucleic Acid Isolation Kit (Life Technologies). RNA quality and quantity were analyzed with Bioanalyzer 2100 and RNA Nano Chips (Agilent Technologies). We have followed RNA extraction protocols that have previously been optimized for FF and FFPE tissues wherein the use of different extraction protocols in a comparative miRNA study was shown to result in expression profiles that are highly reproducible and strongly correlated between FF and FFPE tissue types [24, 84].

### Small RNA library preparation, sequencing and generation of .bam files

Services from PlantBiosis Ltd (Lethbridge, Alberta, Canada; http://www.plantbiosis.com/) were utilized for the preparation of small RNA libraries and for the generation of .bam files. Small RNAs were sequenced using TruSeq Small RNA Sequencing Kit (Illumina), TruSeq SR Cluster Kit v5-CS-GA (Illumina) and TruSeq SBS Kit v5-GA (Illumina) according to manufacturer’s instructions. All the samples were sequenced on Illumina Genome Analyzer IIx with 36-cycle single-end protocol. Base calling and demultiplexing were done using CASAVA 1.8.2 with default settings, followed by trimming of adapters using CutAdapt software (http://code.google.com/p/cutadapt/). Options were given to retain sequences longer than 17 nucleotides. Quality trimming was performed to retain only reads with a Sanger quality score cut-off of 30. The quality of the sequenced reads after adapter trimming was assessed using FASTQC software (http://www.bioinformatics.babraham.ac.uk/projects/fastqc/). One tumor sample was not processed further due to poor quality and was therefore excluded, leaving 103 tumor samples for further analysis. Trimmed sequences were then aligned to the reference genome using Bowtie [85] and were allowed a maximum of two mismatches. Human hg19 genomic assembly (UCSC), downloaded from Illumina iGenome repository was used as a reference for mapping. Aligned sequences were saved as .sam files, converted to more memory efficient .bam files and sorted by genomic position. Sequencing data and the normalized data for miRNAs were submitted to Gene Expression Omnibus (GEO accession ID GSE68085).

### Analysis of .bam files and identification of differentially expressed miRNAs

The .bam files of 11 FF and 103 FFPE tissues were imported into in-house Partek Genomics Suite 6.6 (PGS) software for further analysis (Partek® Genomics Suite software, Version 6.6 beta, Copyright © 2009 Partek Inc., St. Louis, MO, USA). They were mapped to known mature miRNAs using miRBase V20. miRNAs were first filtered to retain only those with a minimum of 10 read counts in at least 90 % of the samples (normal and tumor). Reads per kilobase per million method (RPKM) was used for normalization [86], followed by batch effects correction using the ANOVA model. Principal Component Analysis (PCA) using batch-effects-corrected raw counts was used to identify potential sample outliers. Subsequently, differentially expressed (DE) miRNAs with a fold change (FC) >2.0 and a False Discovery Rate (FDR) cut-off of 0.05 were identified using one-way ANOVA from batch-effects-corrected and filtered normalized counts (after removing sample outliers).

### Statistical considerations for the identification of miRNAs as prognostic markers for BC

*Case–control approach (CC)*: miRNAs were filtered to retain only those with a minimum of 10 read counts in at least 90 % of the tumor and normal samples. Considered as continuous variables, DE miRNAs were subjected to univariate Cox proportional hazards regression model with permutation test (*n* = 10,000), using the R statistical program (‘glmperm’ package) for both OS and RFS. OS and RFS were calculated from the date of diagnosis until an event occurred (death and relapse, respectively). Further statistical analyses were done using SAS (SAS institute Inc., Cary, NC) Version 9.3. For all the statistical analyses except permutation test, p < 0.05 was considered to be statistically significant. The risk score for each sample was constructed for OS and RFS separately, using miRNAs significant in the permutation test (p ≤ 0.1) for OS and RFS. Risk score is a summation obtained by multiplying the expression values of each miRNA with its corresponding co-efficient [18]. In order to dichotomize the cases into low- and high-risk groups, Receiver Operating Characteristic (ROC) curve was used to determine the optimal cut-off point. Kaplan-Meier plots were constructed to estimate the median survival function, and log-rank tests were performed to compare the survival curves of the two survival groups (low- and high-risk groups). Furthermore, multivariate Cox proportional hazards regression model was employed to investigate whether the risk score was a potential independent prognostic factor after accounting for the following variables: age at diagnosis (continuous), tumor stage (I, II vs. III, IV), tumor grade (High vs. Low) and TNBC status (Triple Negative vs. Luminal). Luminal A, Luminal B and Luminal HER2 were grouped as Luminal. Hazard ratio (HR) and their corresponding 95 % confidence interval (CI) were reported for the univariate and multivariate Cox’ regression model.

*Case-only approach (CO)*: miRNAs were filtered to retain only those with a minimum of 10 read counts in at least 90 % of the tumor samples. The filtered list of miRNAs was subjected to univariate analysis (with permutation test), risk score computation and multivariate tests, similar to the case–control approach.

Survival analysis for case–control and case only approaches was performed using only the outcome data from tumor samples. The overall workflow of the study is depicted in Fig. 1.

### qRT-PCR validation of select miRNAs

We have validated three down-regulated miRNAs whose FC ranged from −1.3 to −5.8 and one miRNA which was found to be up-regulated (FC = 12.8), using samples for which RNA was available following NGS. This was done to exemplify the dynamic range of detection and concordance between NGS and qRT-PCR. This analysis included two representative miRNAs (miR-574-3p and miR-660-5p) that were identified to be of prognostic value and considered as novel in BC. miR-99b-5p (FC = −2.3), miR-574-3p (FC = −5.8), miR-769-5p (FC = −1.3) and miR-660-5p (FC = 12.8) were validated using miScript II RT kit (QIAGEN), miScript SYBR Green PCR kit (QIAGEN) and their corresponding miScript Primer Assays according to manufacturers' instructions. All assays were performed in triplicates and human RNU6-2 (QIAGEN) served as the loading control. Fold-expression changes of miRNAs were calculated using the 2^-ΔΔCt^ method [87].

### Breast tumor transcriptome analysis (mRNA) and identification of targets for miRNAs

Messenger RNA (mRNA) expression datasets generated previously (GEO accession ID GSE22820) using Agilent microarray platform were available in-house for 176 tumor samples and 10 normal (reduction mammoplasty) samples. Of these, all the normal samples (*n* = 10) and 17 tumor samples matching with our discovery cohort were selected for analysis. Raw intensity values were Quantile normalized and log 2 transformed, and one-way ANOVA was performed to identify DE genes with FC > 2.0 and FDR cut-off of 0.05 (PGS 6.6).

mRNA targets for miRNAs associated with OS and RFS were first predicted *in silico* using TargetScan database (Version 6.2) (http://www.targetscan.org/). The targets thus obtained were overlapped with DE mRNAs generated from the in-house dataset. The benefit of using mRNA datasets from breast tissues is that they act as a proxy to the functional validation of mRNA targets identified by the *in silico* prediction algorithm. The identification of targets was not restricted to those exhibiting inverse relationships with miRNAs, but any correlation of miRNA to mRNA was captured since the miRNA-mRNA interactions are more complex. Gene ontology (GO) terms were identified for targets of every miRNA separately using DAVID bioinformatics tools v6.7 (http://david.abcc.ncifcrf.gov/) [88]. Only clusters with enrichment scores (ES) ≥ 1.3 [88] were used to identify specific GO terms related to cancer with p < 0.05.

### External validation cohort

A total of 1,088 BC cases were available in The Cancer Genome Atlas (TCGA) and samples were filtered based on the following criteria: (i) female patients, (ii) no history of other malignancy, (iii) samples with no metastasis at the time of diagnosis, (iv) samples from non Caucasian subjects were removed (self declared ethnicity) and (v) invasive ductal carcinoma. A total of 479 samples were retained after filtering for the above criteria and subtype classification data based on the receptor status is available for 332 samples:203 Luminal A, 58 Luminal B, 52 TNBC and 19 HER2+. Of the 332 samples, information on tumor stage was available for 328 samples. TCGA dataset has samples sequenced in Illumina Genome analyzer and in Illumina HiSeq. Of the 328 samples, 156 were sequenced using the former whereas 172 were sequenced using the latter. Only samples sequenced using Illumina HiSeq were considered for this study as the number of samples with events (deaths) were higher in this subset. Since the discovery set did not have Her2+ subtype, we did not consider these samples from the TCGA dataset as well leaving the sample size at 162. We considered a follow up period of > 3 years for patients who were alive based on our previous work to define the minimum follow-up period [81] for recurrence or survival analysis. Overall, a total of 84 samples (with 27 events and 57 patients who were alive) were retained for survival analysis (Table 1). The percent distribution of tumor cells (cellularity) in TCGA dataset were as follows: (i) 30 – 50 % = 14, (ii) 55 – 70 % = 19 and (iii) 75 – 100 % = 50. One sample did not have any information on tumor cellularity. Compared to the TCGA samples, our discovery cohort had all 104 samples at >70 % cellularity and these differences along with NGS platforms utilized for different cohorts should be taken in to account for finer interpretations of the data.

We analyzed .bam files of 84 samples using PGS and were normalized using RPKM method. The normalized counts were adjusted for confounders such as Batch ID, plate ID and Tissue source site (data not shown). Normalized counts for the eleven miRNAs which were significant for OS in CO approach were extracted for constructing the risk score and an optimal cut-off point was determined using ROC. The new risk score was subjected to univariate and multivariate Cox proportional hazards regression model and was adjusted for tumor stage, age at diagnosis and TNBC status. Since the information on recurrence was not available or was limited, we did not consider validation of miRNAs identified for RFS.

## Additional files

Additional file 1: Figure S1. Batch effects correction. Description: 104 tumor and 11 normal samples were sequenced in different batches. ANOVA model was used to capture the different sources of variation. All the factors having a mean F ratio above the mean F ratio of the error bar has to be corrected for (Figure S1a). Since normal and tumor tissues are sources of biological variation, the tissue factor was not corrected for and only batch (being a technical variation) was corrected for. The value of 0 for the factor batch in Figure S1b indicates that the data has been adjusted for batch effects (implemented in Partek Genomics Suite 6.6; see methods). Tissue = Normal and Tumor tissue; Batch = Different batches in which the samples were sequenced. (PDF 39 kb) Additional file 2: Table S1. Differentially expressed miRNAs. Description: miRNAs were profiled from apparently healthy normal (*n* = 11) reduction mammoplasty breast tissues and breast tumor tissues (*n* = 104). RNAs were filtered for low read counts (minimum 10 read counts in at least 90 % of the samples). Following batch effects correction, sample outlier removal and RPKM normalization, differentially expressed RNAs with fold change > 2.0 and a false discovery rate (FDR) < 0.05 were identified. (PDF 63 kb) Additional file 3: Table S2. Gene ontology terms and associated genes. The identified miRNAs significant for OS and RFS (*n* = 12) from both the approaches were interrogated for mRNA targets, followed by identification of Gene ontology terms. Eight out of the 12 miRNAs had targets involved in cell growth and development (p < 0.05). (PDF 102 kb)

## Abbreviations

BC: Breast cancer
CC: Case–control
CO: Case-only
DE: Differentially expressed
ER: Estrogen receptor
ES: Enrichment score
FC: Fold change
FDR: False discovery rate
FF: Fresh frozen
FFPE: Formalin fixed paraffin embedded
GO: Gene ontology
HER2: Human epidermal growth factor receptor 2
HR: Hazard ratio
miRNAs: microRNAs
mRNA: messenger RNA
NGS: Next generation sequencing
OS: Overall survival
PCA: Principal component analysis
PGS: Partek genomics suite
PR: Progesterone receptor
RFS: Recurrence free survival
ROC: Receiver operating characteristic curve
RPKM: Reads per kilobase per million
TCGA: The cancer genome atlas
TNBC: Triple negative breast cancer
