# Supplementary figures and images for: Next generation sequencing profiling identifies miR-574-3p and miR-660-5p as potential novel prognostic markers for breast cancer
Source: BMC Genomics. 2015 Sep 29;16:735. doi: 10.1186/s12864-015-1899-0 (PMC4587870; doi:10.1186/s12864-015-1899-0)

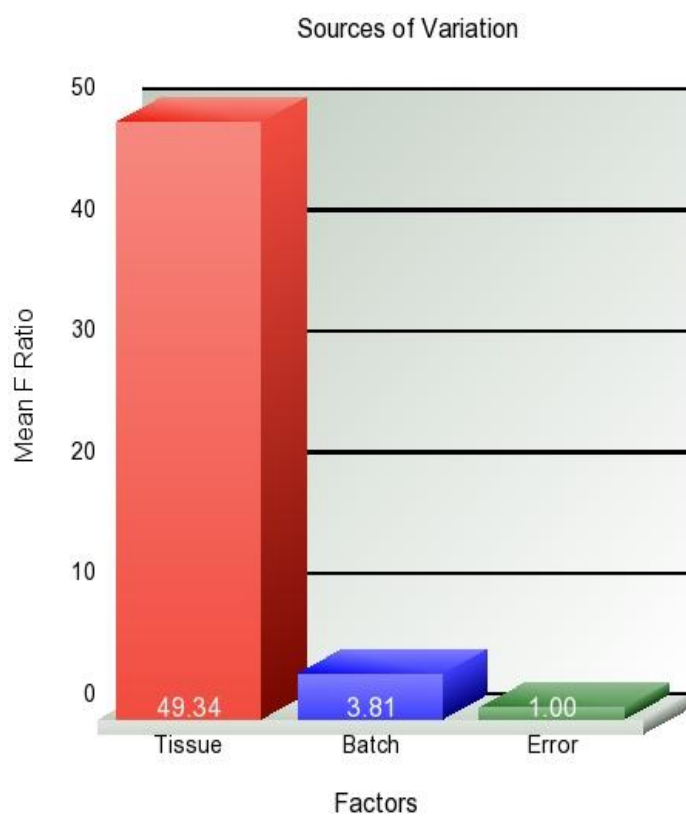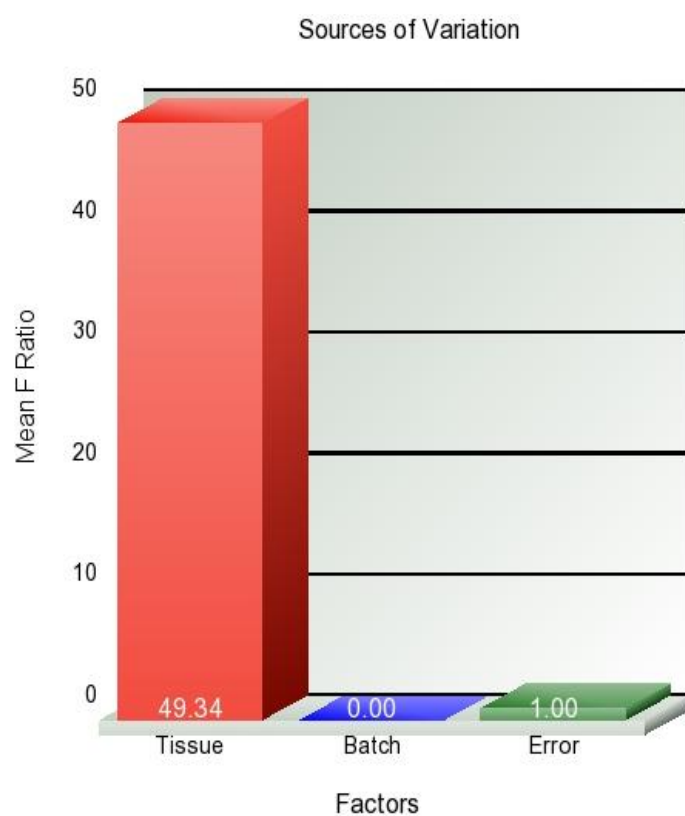

Supplement: Additional file 1: Figure S1. — Batch effects correction. Description: 104 tumor and 11 normal samples were sequenced in different batches. ANOVA model was used to capture the different sources of variation. All the factors having a mean F ratio above the mean F ratio of the error bar has to be corrected for (Figure S1a). Since normal and tumor tissues are sources of biological variation, the tissue factor was not corrected for and only batch (being a technical variation) was corrected for. The value of 0 for the factor batch in Figure S1b indicates that the data has been adjusted for batch effects (implemented in Partek Genomics Suite 6.6; see methods). Tissue = Normal and Tumor tissue; Batch = Different batches in which the samples were sequenced. (PDF 39 kb) [file 12864_2015_1899_MOESM1_ESM.pdf]
