# Supplementary material for: Next generation sequencing profiling identifies miR-574-3p and miR-660-5p as potential novel prognostic markers for breast cancer
Source: BMC Genomics. 2015 Sep 29;16:735. doi: 10.1186/s12864-015-1899-0 (PMC4587870; doi:10.1186/s12864-015-1899-0)
Supplement: Additional file 2: Table S1. — Differentially expressed miRNAs. Description: miRNAs were profiled from apparently healthy normal (n = 11) reduction mammoplasty breast tissues and breast tumor tissues (n = 104). RNAs were filtered for low read counts (minimum 10 read counts in at least 90 % of the samples). Following batch effects correction, sample outlier removal and RPKM normalization, differentially expressed RNAs with fold change > 2.0 and a false discovery rate (FDR) < 0.05 were identified. (PDF 63 kb) [file 12864_2015_1899_MOESM2_ESM.pdf]

miRNA ID	p-value	FDR	Fold Change	Direction of Fold change
hsa-let-7a-5p	2.32E-20	1.35E-19	-3.71714	Down-regulated in tumor
hsa-let-7b-3p	2.00E-32	3.10E-31	-12.9996	Down-regulated in tumor
hsa-let-7b-5p	9.92E-21	6.59E-20	-4.77593	Down-regulated in tumor
hsa-let-7c-5p	8.16E-30	9.62E-29	-8.04453	Down-regulated in tumor
hsa-let-7d-3p	1.04E-29	1.07E-28	-16.2072	Down-regulated in tumor
hsa-let-7d-5p	9.82E-09	2.77E-08	-2.20639	Down-regulated in tumor
hsa-let-7e-5p	1.98E-06	3.68E-06	-2.20181	Down-regulated in tumor
hsa-let-7f-5p	1.35E-08	3.58E-08	5.96238	Up-regulated in tumor
hsa-let-7i-5p	5.18E-05	8.03E-05	2.9048	Up-regulated in tumor
hsa-miR-101-3p	2.71E-06	4.84E-06	5.0085	Up-regulated in tumor
hsa-miR-103a-3p	9.08E-08	2.06E-07	6.93112	Up-regulated in tumor
hsa-miR-107	1.76E-07	3.72E-07	10.8589	Up-regulated in tumor
hsa-miR-10a-5p	0.0347755	0.0347755	2.41909	Up-regulated in tumor
hsa-miR-125a-5p	5.30E-37	1.23E-35	-9.59744	Down-regulated in tumor
hsa-miR-125b-5p	4.12E-29	3.83E-28	-4.909	Down-regulated in tumor
hsa-miR-126-3p	0.0008268	0.0010534	3.04256	Up-regulated in tumor
hsa-miR-126-5p	0.0002196	0.0003094	12.2333	Up-regulated in tumor
hsa-miR-141-3p	0.015057	0.0159125	4.03387	Up-regulated in tumor
hsa-miR-142-5p	0.0175575	0.0183466	26.742	Up-regulated in tumor
hsa-miR-145-5p	3.05E-19	1.49E-18	-4.44216	Down-regulated in tumor
hsa-miR-148a-3p	0.0014697	0.0017984	5.75801	Up-regulated in tumor
hsa-miR-148a-5p	0.0044288	0.0050849	-2.00773	Down-regulated in tumor
hsa-miR-150-5p	5.96E-09	1.73E-08	-3.8121	Down-regulated in tumor
hsa-miR-151a-3p	0.0001104	0.0001604	2.99004	Up-regulated in tumor
hsa-miR-155-5p	0.0304092	0.0310775	2.53284	Up-regulated in tumor
hsa-miR-15a-5p	4.24E-08	1.04E-07	12.1568	Up-regulated in tumor
hsa-miR-15b-5p	9.93E-18	4.40E-17	-2.91538	Down-regulated in tumor
hsa-miR-16-5p	3.00E-11	1.12E-10	27.2819	Up-regulated in tumor
hsa-miR-17-5p	0.0049192	0.0055791	3.57987	Up-regulated in tumor
hsa-miR-181a-2-3p	6.09E-05	9.28E-05	-2.04284	Down-regulated in tumor
hsa-miR-181a-5p	1.70E-09	5.27E-09	-2.35667	Down-regulated in tumor
hsa-miR-181d-5p	0.0049893	0.0055905	3.80132	Up-regulated in tumor
hsa-miR-182-5p	0.0287213	0.0296787	33.0627	Up-regulated in tumor
hsa-miR-183-5p	0.0058226	0.0064464	9.66818	Up-regulated in tumor
hsa-miR-192-5p	0.0031425	0.0037468	2.93814	Up-regulated in tumor
hsa-miR-193b-3p	3.25E-15	1.37E-14	-4.338	Down-regulated in tumor
hsa-miR-195-5p	0.0036828	0.0042812	2.86077	Up-regulated in tumor

hsa-miR-196b-5p	0.0133398	0.0142598	57.9456	Up-regulated in tumor
hsa-miR-197-3p	6.00E-41	2.79E-39	-13.9857	Down-regulated in tumor
hsa-miR-199a-3p	2.53E-05	4.15E-05	13.2278	Up-regulated in tumor
hsa-miR-199a-5p	0.0003248	0.0004442	5.4778	Up-regulated in tumor
hsa-miR-199b-3p	2.55E-05	4.15E-05	13.3249	Up-regulated in tumor
hsa-miR-199b-5p	0.000889	0.0011173	3.65272	Up-regulated in tumor
hsa-miR-19b-3p	1.12E-08	3.07E-08	3.36478	Up-regulated in tumor
hsa-miR-200b-3p	0.0111238	0.0121707	3.75366	Up-regulated in tumor
hsa-miR-205-5p	0.0004903	0.0006343	-2.48868	Down-regulated in tumor
hsa-miR-20a-5p	0.0001847	0.0002642	17.929	Up-regulated in tumor
hsa-miR-21-3p	0.0025673	0.0031008	10.1748	Up-regulated in tumor
hsa-miR-214-3p	4.35E-27	3.11E-26	-5.77584	Down-regulated in tumor
hsa-miR-21-5p	5.84E-08	1.39E-07	3.87058	Up-regulated in tumor
hsa-miR-22-3p	0.0013836	0.0017157	2.4562	Up-regulated in tumor
hsa-miR-24-3p	3.50E-05	5.52E-05	2.43084	Up-regulated in tumor
hsa-miR-26b-5p	8.99E-07	1.74E-06	32.233	Up-regulated in tumor
hsa-miR-27a-3p	6.42E-08	1.49E-07	6.4593	Up-regulated in tumor
hsa-miR-27b-3p	2.23E-06	4.07E-06	4.34738	Up-regulated in tumor
hsa-miR-28-3p	5.06E-06	8.71E-06	-2.04801	Down-regulated in tumor
hsa-miR-28-5p	0.033187	0.0335477	2.1824	Up-regulated in tumor
hsa-miR-29c-3p	7.34E-07	1.45E-06	2.89628	Up-regulated in tumor
hsa-miR-30a-5p	0.0124884	0.0135049	12.8942	Up-regulated in tumor
hsa-miR-30e-5p	1.23E-06	2.34E-06	24.3863	Up-regulated in tumor
hsa-miR-320a	6.53E-45	6.07E-43	-7.08391	Down-regulated in tumor
hsa-miR-335-5p	0.0004911	0.0006343	21.7075	Up-regulated in tumor
hsa-miR-340-5p	1.11E-09	3.56E-09	2.5164	Up-regulated in tumor
hsa-miR-342-3p	0.0003624	0.0004885	6.19359	Up-regulated in tumor
hsa-miR-34a-5p	1.93E-05	3.27E-05	13.3181	Up-regulated in tumor
hsa-miR-378a-3p	9.60E-05	0.0001417	-2.31521	Down-regulated in tumor
hsa-miR-409-3p	3.47E-12	1.34E-11	-3.5944	Down-regulated in tumor
hsa-miR-423-3p	1.79E-35	3.33E-34	-5.45872	Down-regulated in tumor
hsa-miR-423-5p	9.69E-39	3.00E-37	-11.2111	Down-regulated in tumor
hsa-miR-429	0.000259	0.0003595	2.46338	Up-regulated in tumor
hsa-miR-486-5p	1.31E-20	8.13E-20	-15.2202	Down-regulated in tumor
hsa-miR-497-5p	1.86E-07	3.84E-07	-2.78244	Down-regulated in tumor
hsa-miR-574-3p	8.28E-30	9.62E-29	-5.80108	Down-regulated in tumor
hsa-miR-654-3p	4.82E-10	1.66E-09	-3.64477	Down-regulated in tumor
hsa-miR-660-5p	7.13E-07	1.44E-06	12.8884	Up-regulated in tumor
hsa-miR-92a-3p	2.22E-28	1.88E-27	-5.3314	Down-regulated in tumor

hsa-miR-92b-3p	8.75E-10	2.91E-09	-3.14854	Down-regulated in tumor
hsa-miR-93-5p	0.003216	0.0037859	3.53509	Up-regulated in tumor
hsa-miR-98-5p	1.11E-07	2.40E-07	22.146	Up-regulated in tumor
hsa-miR-99b-5p	1.36E-12	5.50E-12	-2.32803	Down-regulated in tumor
