# Supplementary material for: Next generation sequencing profiling identifies miR-574-3p and miR-660-5p as potential novel prognostic markers for breast cancer
Source: BMC Genomics. 2015 Sep 29;16:735. doi: 10.1186/s12864-015-1899-0 (PMC4587870; doi:10.1186/s12864-015-1899-0)
Supplement: Additional file 3: Table S2. — Gene ontology terms and associated genes. The identified miRNAs significant for OS and RFS (n = 12) from both the approaches were interrogated for mRNA targets, followed by identification of Gene ontology terms. Eight out of the 12 miRNAs had targets involved in cell growth and development (p < 0.05). (PDF 102 kb) [file 12864_2015_1899_MOESM3_ESM.pdf]

Gene ontology term	miRNA ID	Gene targets
Transcription	hsa-miR-660-5p	EGR2, NPAS3, ZBTB34, RFX4, EPAS1, NFAT5, ETV1, NR3C1, MEIS1
	hsa-miR-374a-3p; hsa-miR-374a-5p	CEBPA, HLF, ZBTB34, EGR2, BACH2, RFX4, EPAS1, ARID5B, ONECUT2, TLE4, CREB5, NFIX, NEUROG2, NR3C1, TCEAL7, LMX1A, MEIS1, EBF3, PNRC1, GBX2, HOXA10, SHOX, NFIB
	hsa-miR-221-3p	CDKN1C, SOX10, FOS, NTF3, GATA4, HOXA7, NFAT5, IGF1, GLI2, FOXP2, ZFP36, ZFP36L2, EIF4E3, QKI, TNRC6B
	hsa-miR-196a-5p	ING5, ERG, ZNF516, HOXA5, E2F7, BCL11A, HOXA7, HOXA9, TGFBR3, HABP4, IGF1, HMGA2, LIN28B, FOXP2
	hsa-miR-27a-3p	MEF2C, ING5, ZNF516, ZBTB34, E2F7, PPARG, ONECUT2, SOX7, EHF, PRDM16, SOX8, NPAS3, HOXA5, NR1D2, BCL11A, NFAT5, HOXA10, ERG, SMAD9, RUNX1T1, PPARGC1B, NRIP2, FOXP2, FOXN4, ZFHX4, HOXC11, ATF3, EBF3, BCORL1, NEUROD4, CAND1, NFIB
Cell morphogenesis	hsa-miR-374a-3p; hsa-miR-374a-5p	BMP2, EGR2, NTF3, ONECUT2, NEUROG2, L1CAM, LMX1A, SLIT3, SEMA5A, EPHA4, DMD, GBX2, CNTN4
	hsa-miR-221-3p	NTF3, PVRL1, DCX, GLI2, CXCL12
	hsa-miR-27a-3p	EGFR, SEMA6A, MAP1B, PRICKLE2, ONECUT2, LIFR, TGFBR3, RELN, NRXN1, NGFR, DCX, CACNA1A
Cell motion	hsa-miR-374a-3p; hsa-miR-374a-5p	SEMA5A, EPHA4, EGR2, NTF3, ARID5B, GBX2, NEUROG2, L1CAM, CNTN4, LMX1A, PPAP2B, SLIT3
	hsa-miR-221-3p	NTF3, PVRL1, WASF2, EMX2, IGF1, KIT, DCX, GLI2, CXCL12

	hsa-miR-196a-5p	PDGFRA, TGFBR3, IGF1, SEMA3A
	hsa-miR-27a-3p	RET, MET, IGF1, NRXN1, COL5A1, SEMA6A, BTG1, TGFBR3, NEUROD4, RELN, NGFR, DCX, PPAP2B
	hsa-miR-15a-5p	BDNF, PVRL1, PODXL, TGFBR3, IGF1, RELN, SEMA3A, LAMC1, CX3CL1, PPAP2A, FGF2, PPAP2B
Angiogenesis	hsa-miR-374a-3p; hsa-miR-374a-5p	SEMA5A, EPAS1, FGF9, GBX2, TGFA
	hsa-miR-15a-5p	RTN4, MEOX2, FGF9, PLCD1, FGF1, FIGF, FGF2
Cell migration	hsa-miR-221-3p	PDGFA, IGF1, KIT, CXCL12, PIK3R1
	hsa-miR-27a-3p	RET, BTG1, MET, TGFBR3, NEUROD4, RELN, DCX, PPAP2B, COL5A1
	hsa-miR-15a-5p	PODXL, TGFBR3, RELN, LAMC1, CX3CL1, PPAP2A, FGF2, PPAP2B
Cell proliferation	hsa-miR-221-3p	ZFP36L2, PDGFA, EMX2, IGF1, KIT, NRG1, GLI2, CXCL12
	hsa-miR-196a-5p	TGFBR3, IGF1, FOXP2
	hsa-miR-15a-5p	TXNIP, FGFR1, FGF7, FGF9, E2F7, IGF1, FOXP2, PTHLH, BDNF, TRIM35, TBRG1, TGFBR3, ADAMTS1, RARB, LAMC1, AXIN2, PPAP2A, FGF1, NRG1, FIGF, FGF2, HTR2A
Cell signaling	hsa-miR-221-3p	NTF3, PVRL1, PDGFA, FGF14, GATA4, KCNA1, CACNB4, CXCL11, GLI2, NOVA1
	hsa-miR-27a-3p	STX1A, FGF14, CACNB2, NRXN1, GRIA4, LEP, SPRY2, ECE2, PDE7B, WISP1, HOXC11, FGF1, CACNA1A, NOVA1, DTNA
	hsa-miR-15a-5p	STX1A, KCNC4, FGF9, NLGN1, PTHLH, BDNF, WISP1, KIF1B, HOXC11, PVRL1, GRM7, FGF1,

		CHRNE, FGF2, HTR2A
Blood vessel development	hsa-miR-221-3p<br><br>hsa-miR-15a-5p	RECK, PDGFA, WASF2, QKI, CXCL12<br><br>RTN4, RECK, MEOX2, FGF9, TGFBR3, QKI, PLCD1, FGF1, FIGF, FGF2, PPAP2B
Cytoskeleton organization	hsa-miR-146b-5p	PRC1, WASF3, TLN2, WASF2, ABL2
Response to estrogen stimulus	hsa-miR-27a-3p	PPARG, MAP1B, PDGFRA, MMP13, CCNA2
Positive regulation of cell differentiation	hsa-miR-27a-3p	LEP, LPL, ACVR2A, SMAD9, BTG1, CSF1, PPARG, MAP1B, NGFR
Regulation of cell division	hsa-miR-15a-5p	FGF7, FGF9, FGF1, FIGF, FGF2
Regulation of cell growth	hsa-miR-15a-5p	RTN4, EXTL3, WISP1, TSPYL2, SEMA3A, NRG1, FGF2, CRIM1
Mammary gland development	hsa-miR-221-3p	IGF1, NRG1, GLI2
Mesenchymal cell differentiation	hsa-miR-374a-3p; hsa-miR-374a-5p	BMP2, GBX2, CYP26A1
